# CRISPR/Cas9-Mediated Gene Editing in Salmonids Cells and Efficient Establishment of Edited Clonal Cell Lines

**DOI:** 10.3390/ijms232416218

**Published:** 2022-12-19

**Authors:** Trygve A. H. Strømsnes, Sebastian E. Schmidke, Mitra Azad, Øyvind Singstad, Idun M. Grønsberg, Roy A. Dalmo, Arinze S. Okoli

**Affiliations:** 1 NORCE Norwegian Research Centre AS, Climate & Environment Division, Siva Innovasjonssenter, Sykehusveien 21, 9019 Tromsø, Norway; 2 Norwegian College of Fishery Science, Faculty of Biosciences, Fisheries and Economics, UiT—The Arctic University of Norway, Muninbakken 21, 9019 Tromsø, Norway

**Keywords:** CRISPR/Cas9, A. salmon, gene editing, ASK-1, SHK-1, CHSE-214, plasmid, ribonucleoprotein, aquaculture

## Abstract

Finfish production has seen over three-fold increase in the past 30 years (1990–2020), and Atlantic salmon (A. salmon; *salmo salar*) accounted for approximately 32.6% of the total marine and coastal aquaculture of all finfish species in the year 2020, making it one of the most profitable farmed fish species globally. This growth in production is, however, threatened by a number of problems which can be solved using the CRISPR/Cas technology. In vitro applications of CRISPR/Cas using cell lines can complement its in vivo applications, but salmonids-derived cell lines are difficult to gene edit because they grow slowly, are difficult to transfect and isolate single clones of gene-edited cells. While clonal isolation of the gene-edited Chinook salmon cell line (CHSE-214) has successfully been performed, there is no report of successful clonal isolation of the gene-edited A. salmon ASK-1 and SHK-1cell lines. In the current study, two gene loci—*cr2* and *mmp9* of A. salmon—were efficiently edited using the ribonucleoprotein (RNP) and plasmid CRISPR/Cas9 strategies. Edited cells were enriched using flow cytometer-activated cell sorting (FACS), followed by clonal isolation and expansion of edited cells. The study both confirms the recent report of the highly efficient editing of these widely used model cell lines, as well as extends the frontline in the single-cell cloning of gene-edited salmonids cells. The report also highlights the pitfalls and future directions in the application of CRISPR/Cas9 in these cells.

## 1. Introduction

World aquaculture production is at an all-time high, generating 122.6 million tons in 2020 valued at USD 281.5 billion, and estimated to reach 202 million tons in 2030 [1]. Of this, the contribution of fisheries (excluding algae) reached 49.2% compared to 13.4% in 1990, with finfish production dwarfing all other fish species with a 46.9% share in world fish production [1]. The share of Atlantic salmon (A. salmon; *salmo salar*) in the total finfish production was 2.7 million tons, accounting for 32.6% of marine and coastal aquaculture of all finfish species in the year 2020 [1], and making A. salmon one of the most successful and profitable farmed fish species globally. Thus, the salmonid fish species, including A. salmon, is rapidly becoming one of the world’s greatest sources of protein. This expansion in farmed fish aquaculture, in particular A. salmon breeding, is threatened by a number of problems including outbreaks of infectious diseases, reduced viability, escapee fish, slow growth and fertility reduction [2,3]. Gene editing, especially CRISPR/Cas9 (clustered regularly interspaced short palindromic repeats (CRISPR)/CRISPR associated protein 9), in combination with targeted breeding programmes, can be a valuable tool to solve these problems and enhance the sustained growth of the industry as this technique allows targeted changes to the genomes of species of interest; can be applied to all cells and organisms including non-model species such as A. salmon; and is efficient, cheap and relatively easy to use. Technically, CRISPR/Cas9 can be applied to specifically edit any gene of any organism. Its main components are (i) 20–22 base pair (bp) CRISPR single-guide RNA (sgRNA) and (ii) endonuclease enzyme, Cas9. The sgRNA can be engineered to target any gene. Fusion or amalgamation of the sgRNA and the Cas9 results in a conformational change in the Cas9 leading to a double-strand break (DSB) at a specific DNA region homologous to the sgRNA. Cellular repair of the DSB often leads to deletion and/or insertion (indel) mutations, thus, editing the target gene [4,5]. 

CRISPR/Cas9, which is already revolutionizing the fields of agriculture [6], human [7] and veterinary medicines [8], is also being applied to aquaculture with tremendous success. For example, the technology has successfully been applied to experimentally edit the genome of A. salmon producing sterile salmon [9], albino salmon [10] and an A. salmon with potential for increased omega-3 production [11]. Further, CRISPR/Cas9-edited growth-enhanced tiger puffer fish and a red sea bream fish are now commercially available [12]. Gene editing can also be used, in combination with selective breeding, for improved host resistance against major fish viral [13], parasitic [14] and bacterial diseases [15]. The in vitro application of CRISPR/Cas9 in fish cell lines lags behind its in vivo application in the fish organism, but studies using engineered cell lines can complement in vivo investigation to elucidate functional mechanisms that underly, for example, salmonids response to pathogens. In vitro gene editing in cell lines will allow for optimization of methods before in vivo testing, saving time and costs in experiments, as well as minimizing the number of fish to be used in an experiment. Further, it is necessary to conduct gene editing in cell lines before in vivo trials in order to ascertain whether a knockout will be lethal and or induce undesired effects, which can impact fish welfare. For example, assessment and optimization of the efficiencies of the various CRISPR/Cas9 strategies against relevant gene targets can be conducted in vitro, allowing for selection of highly prioritized genes for in vivo gene editing. However, salmonids cell lines are difficult to gene edit because of the difficulty in transfecting these cell lines, slow growth characteristics of the cells, and difficulty developing clonal cell lines. In addition, the currently available CRISPR/Cas9 toolkits, in particular the Cas9 proteins and CRISPR/Cas plasmid vectors, are not adapted to the incubation conditions, e.g., low temperatures at which salmonids fish cell lines are incubated. For A. salmon, these problems are further compounded by the poor annotation of existing sequenced genomes [16] and the duplication event in fish, in particular finfish species [17], which hinders the clarification of trait-related genes, i.e., which genes should be targeted for gene editing. 

Nevertheless, improvements in CRISPR/Cas9 strategies are steadily helping to overcome these limitations. Recent studies have reported an improved gene editing efficiency of 62% in medaka fish cell line (*Oryzias latipes*) [18] and >90% efficiency in some salmonids-derived cell lines [19] using the ribonucleoprotein (RNP) CRISPR/Cas9 strategy, compared to the 34.6% editing efficiency reported with the first application of the strategy in Chinook salmon (*Oncorhynchus tshawytscha*) [20]. Similarly, steady improvements have been reported in the vector strategies of CRISPR/Cas9 in fish cell lines, although with much lower efficiencies (1–10%) [21,22] compared with the RNP strategy. Further, some progress has been made with respect to the development of gene-edited salmonids clonal cell lines, but the problem persists, especially with A. salmon cells. The first successful report of clonal isolation following gene editing was in the Chinook salmon-derived CHSE-214 cell line and was used to show the response of a *stat2* knockout cell line to viral infections [23]. Recently, establishment of a Rainbow trout (*Oncorhynchus mykiss*)-derived clonal cell line harbouring disruption in the *cyp1a1* gene locus was reported [24] using, however, the rather old and difficult-to-reproduce cloning cylinders after unsuccessful attempts with the FACS (fluorescence-activated cell sorting)-enriched cells. Additionally, an attempt to clonally isolate the A. salmon-derived SHK-1 cell after gene editing was not successful [19], and single cell cloning of the ASK-1 cell line has not been performed. Thus far, and to the best of our knowledge, clonal isolation of gene-edited A. salmon-derived cell lines has not been reported. Single cell cloning is paramount for downstream investigation of the phenotypic relevance of specific gene editing, and for the high-efficiency editing of gene targets, especially for loci with low editing efficiencies. In the current study, the establishment of single clonal cell lines of ASK-1 and CHSE-214 cell lines was achieved after FACS enrichment of RNP gene-edited cells. Furthermore, clonal selection was achieved in the SHK-1 cell line using a combination of plasmid-based CRISPR/Cas9 strategy and antibiotic selection, thus, verifying the recent report on the efficient gene editing of salmonids cells, as well as extending the frontline in the single cell cloning of gene-edited A. salmon cell lines. 

## 2. Results

### 2.1. Efficient sgRNA Synthesis Resulted in Effective In Vitro RNP Cleavage of Target Genes

**Figure 1:**
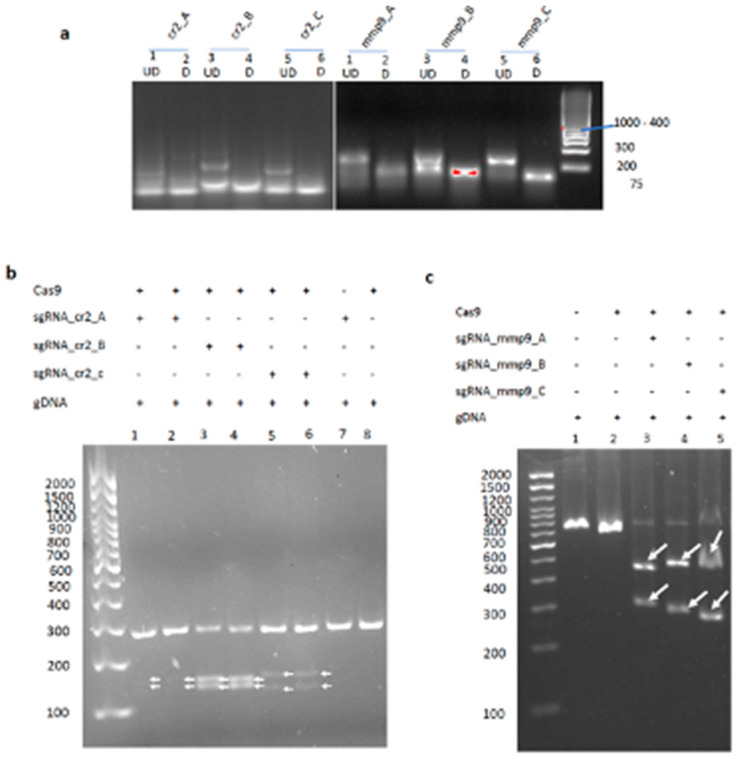
sgRNA analysis and in vitro cleavage assay for *cr2* and *mmp9* genes. (**a**) sgRNA analysis on 2% agarose gel. UD: undenatured sgRNA, D: denatured sgRNA. Single bands detected for the denatured sgRNAs highlighted the purity of the products, and multiple bands in the undenatured sgRNA resulted from the presence of RNA secondary structures; (**b**) gel shows duplicate lanes for each sample. Lanes 1–6: experimental samples, lane 7: no Cas9 control, lane 8: no sgRNA control; (**c**) lane 1: no Cas9 control, lane 2: no sgRNA control, lanes 3–5: experimental samples. Cleavage products at expected band sizes were detected for all samples containing the sgRNA + Cas9 + gDNA. No product at the expected band size was observed for all the control samples.

For quality assurance and as a cost-saving strategy, all sgRNAs used in this study (Table 1) were produced in-house using self-designed target-specific oligos and an optimized sgRNA synthesis protocol. High-yield and high-quality sgRNAs were produced: *cr2* (yield: 554.9–1446 ng/μL; purity: 2.0–2.17 A260/A280 ratio); *mmp9* (yield: 1940–2800 ng/μL; purity: 2.0–2.32 A260/A280 ratio). A gel electrophoresis of heat-denatured and non-heat denatured sgRNAs showed single bands (for the heat-denatured sgRNAs), confirming the purity of the synthesized sgRNAs (Figure 1a). Multiple bands in the non-heat denatured sgRNAs expectedly resulted from intramolecular base-pairing of the RNA secondary structures. 

To determine the ability of the synthesized sgRNAs to guide the Cas9 to the specific loci of *cr2* and *mmp9* genes and cause DNA cleavage, an in vitro cleavage assay was performed. PCR amplicons of the *cr2* (~amplicon size 289 bp) and *mmp9* (~amplicon size 710 bp) gene loci were incubated with sgRNA and Cas9-EGFP (also simply written as Cas9). As expected, cleavage products represented by double bands for each gene, *cr2* (~160 and ~129 bp) and *mmp9* (~420 and ~290 bp), were observed (Figure 1b,c). No cleavage product was detected for the control mixtures containing either sgRNA or Cas9, indicating an effective targeting to the desired loci and cleavage of the gene amplicons by all the synthesized sgRNAs.

### 2.2. Transfection and Enrichment of Cells Following Electroporation with RNP, px458 and px459 

**Figure 2:**
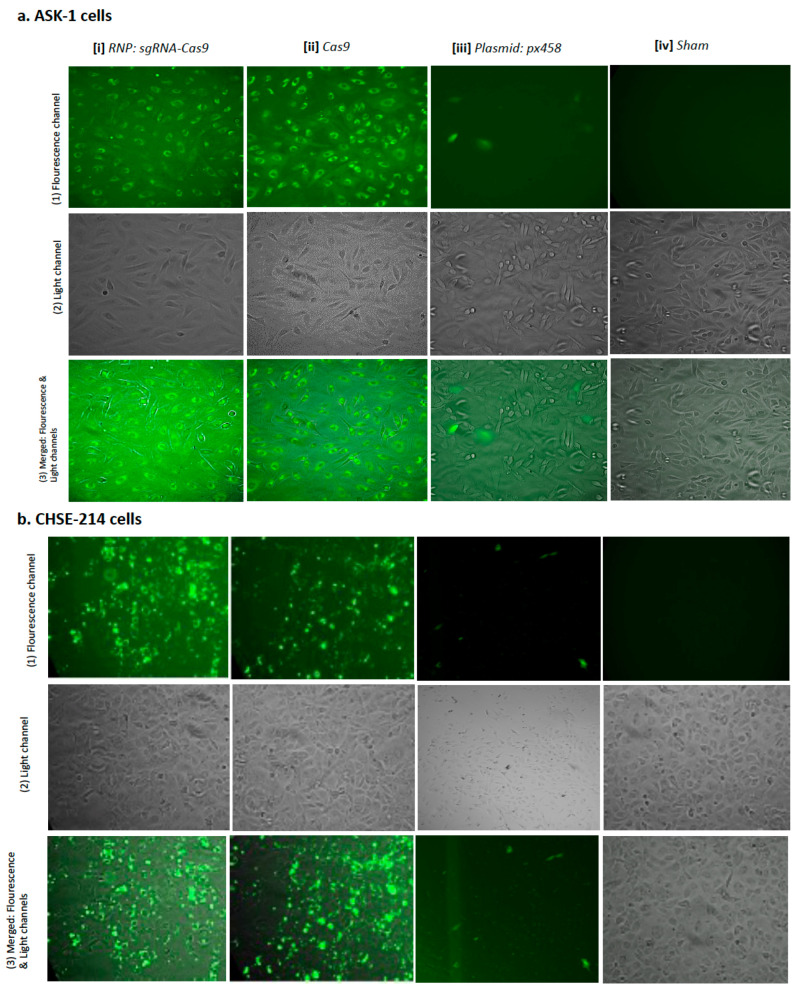
Ribonucleoprotein (RNP) and plasmid (px458) transfection of (**a**) ASK-1 and (**b**) CHSE-214 cells. Vertical panels show cells transfected with (i) RNP (sgRNA:Cas9-EGFP complex); (ii) Cas9-EGFP protein control; (iii) plasmid (px458); (iv) sham control. Horizontal panels show pictures of cells taken with (1) fluorescent channel; (2) light channel; (3) overlay of the light and fluorescent panels. Pictures were taken at day 7 after RNP electroporation (1600 V, 10 ms, 3 pulses) and day 2 after px458 electroporation (1200 V, 20 ms, 2 pulses). Strong adherence of Cas9-EGFP was observed on the cell surface of CHSE-214, and it was difficult to entirely wash off with PBS, explaining the high background fluorescence in the Cas9-EGFP- and RNP-transfected CHSE-214 cells. Magnification X20. (Fluorescent pictures of transfected SHK-1 cells are not shown).

Gene editing of the *cr2* and *mmp9* genes in ASK-1, CHSE-214 and SHK-1 was performed via transfection of the RNP (sgRNA:Cas9 complexes), px458 (encoding Cas9, sgRNA and GFP) and px459 (encoding Cas9, sgRNA and puromycin). It is worth noting that the Cas9 used for the RNP transfection in this study is conjugated with EGFP (enhanced green fluorescent protein). The in-house optimized electroporation parameters for plasmids (px458 and px459) were 1200 V, 20 milliseconds (ms) and 2 pulses, and the previously optimized parameters for RNP [19] were 1600 V, 10 ms and 3 pulses, which were delivered to the RNP/cells or plasmid/cells mixtures. Positive transfection—measured by green fluorescence and flow cytometry for RNP and px458, or by antibiotic selection for px459—of approximately 90–95% was obtained for the RNP-transfected ASK-1, CHSE-214 and SHK-1 cells following electroporation (data for transfected SHK-1 cells not shown), compared to approximately 1–5% efficiency of the px458 electroporated cells (Figure 2). Positive transfection with px459 was only recorded for SHK-1 cells indicated by puromycin selection. 

The fluorescence intensity was independent of the sgRNA:Cas9 ratio, i.e., the intensity was the same for 1:1, 3:1 and 9:1 sgRNA:Cas9 ratios, but was highest between day 4 and day 10 following electroporation with both RNP and Cas9-GFP and was still visible in approximately 70% of the cells after 14 days. For px458 electroporated cells, the highest intensity was observed at day 2 following electroporation and had diminished after 4 days. The fluorescence intensity in the px458-electroporated cells was equally distributed in both the nucleus and cytoplasm. However, in the RNP electroporated cells, the fluorescence intensity was greater in the cytoplasm than the nucleus; this was more noticeable in the ASK-1 cells and less so in the CHSE-214 cells and cells electroporated with only Cas9 (Figure 2a,b). Cas9 strongly adhered to the surface of the CHSE-214 cells and was difficult to wash off with several rounds of PBS washes, resulting in a high background fluorescence for RNP- and Cas9-transfected CHSE-214 cells (Figure 2b). 

The enrichment of the RNP-transfected cells was performed at days 4, 7 and 14 post-electroporation using FACS, and positive px458 transfected cells were enriched at day 2 post electroporation. Due to the strong adherence of Cas9-GFP to the cell surface, especially the surface of the CHSE-214 cells (Figure 2b), both SHAM- and Cas9-GFP electroporated cells were used as background controls and were analysed first during FACS. This served a stringent control that enabled the appropriate setting of gates for the isolation of only fluorescent cells from the RNP electroporated cells. As a result, only an approximate 10% of the strongly positive transfected cells were selected during FACS, from which single positive transfected cells were sorted into 96-well plates, while the remainder were sorted into a well of a 24-well plate. The 24-well plate is a heterogenous cell collection, while the 96-well plate is a homogenous cell collection with respect to the type of gene edit. Both plates contained 200–500 μL of conditioned media without antibiotics and were incubated in the dark at 22 °C. 

### 2.3. Mismatch Assay and Sanger Sequencing Revealed Efficient In Vivo CRISPR/Cas9 Editing of Targeted Genes

**Figure 3:**
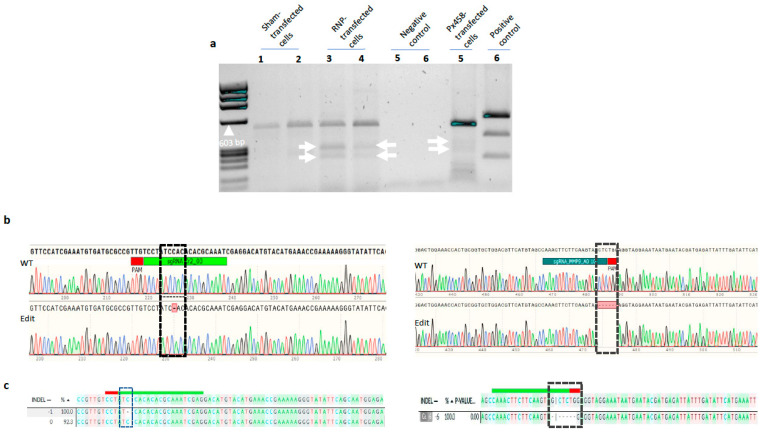
Analyses of mutations in the target regions of *cr2* and *mmp9* genes due to the applied CRISPR/Cas9 strategies. (**a**) T7E1 assay of the *cr2* RNP-transfected CHSE-214 cells. Cleaved fragments corresponding to T7 endonuclease 1 digestion of gDNA extract from the RNP-transfected cells are indicated with white arrows in lanes 3 and 4. Similar results were obtained in RNP-transfected ASK-1 and SHK-1 cells for both *cr2* and *mmp9*. (**b**) Representative Sanger sequencing chromatogram for the target region of *cr2* (upper left) and mmp9 (upper right) in ASK-1 cells, either wild-type (WT) or edited with CRISPR/Cas9. The binding regions are represented by the sgRNA rectangular bar, and the edited regions are boxed in dashed lines. (**c**) Representative output of DECODRE analysis of a *cr2* RNP-transfected- (lower left) and an *mmp9* RNP-transfected (lower right) ASK-1 cell. Indel type and editing frequency are indicated, as well as the aligned sequences of the wild-type and edited samples. The sgRNA including the PAM sequences (red and green bar) highlights the exact target sequence and Cas9 cut site. Boxed dashed lines indicate the exact site of the observed indel mutations.

A qualitative detection of mutation by the CRISPR/Cas9 system in the *cr2* and *mmp9* genes was performed by a T7E1 assay using the gDNA PCR amplicons of RNP- and plasmid-transfected and control (i.e., non-transfected) ASK-1, SHK-1 and CHSE-214 cells. If a mutation is present as a result of the NHEJ (non-homologous end-joining repair system) repair caused by a Cas9 double-strand break (DSB), denaturation and re-annealing of the PCR products will lead to the formation of a heteroduplex and wild-type amplicons. The T7 endonuclease 1 recognizes (indels ≥ 2 bases) and cleaves non-perfectly matched DNA sequences in a heteroduplex. As shown in Figure 3a, low molecular weight DNA bands of the correct size, corresponding to the heteroduplex digestion products, were detected for the *cr2* target region using amplicons from RNP- and px458-transfected CHSE-214 cells. This clearly indicated that gene editing occurred in the gene locus when RNP complexes or px458 were transfected in CHSE-214 cells. The absence of cleavage product in the analysed amplicon from the non-transfected control further confirms the specificity of the applied CRISPR/Cas9 strategies. Similar results were obtained in ASK-1 and SHK-1 cells using either *cr2* or *mmp9* amplicons.

Editing efficiency was determined by deconvolution of the Sanger sequencing data of the amplified target regions of the *cr2* and *mmp9* genes obtained from both the heterogenous FACS-enriched cell collection (in the 24-well plates) and the homogenous clonal single cells derived from 16 randomly selected cells in the FACS-sorted 96-well plates. Comparison between the chromatograms in the two representative samples from the sham-transfected control cells (wild-type, WT) versus the RNP-transfected cells (edit) showed missing chromatograms in the regions targeted by the sgRNAs (Figure 3b), indicating the presence of indels in these targeted regions. Chromatograms of plasmid-transfected versus sham-transfected control cells also showed indel mutations. The editing efficiency of the Cas9 RNP complex was dependent on cell type because an overall editing efficiency of over 93% was achieved in ASK-1 cells but varied between 5–100% in CHSE-214 cells for the same *cr2* target. The editing efficiency was also dependent on the target region, as higher efficiency was recorded for *cr2* than *mmp9* between ASK-1 and CHSE-214. The RNP gene editing strategy was unsuccessful in the SHK-1 cell despite several attempts. The SHK-1 cells did not survive long enough or were lost due to bacteria contamination following FACS enrichment. Gratacap et al. were also unable to achieve clonal expansion of SHK-1 cells, even with the use of antibiotics to protect against bacteria contamination [19]. It is noteworthy that antibiotics were not used in the culture media in the present study. Similarly, gene editing was not successful in the px458-transfected cells as FACS enrichment could not be achieved due to the extremely low success rate (1–5%) of transfection indicated by only a few green fluorescent cells (Figure 2). However, the plasmid (px459) gene editing strategy was successful in the SHK-1 cell with an editing efficiency of 100% for the *cr2* gene target. All experiments were performed in triplicates and repeated at least once, except for the single clonal experiment, which was conducted only once due to the long duration (approximately 9 weeks) from the identification of a single cell-derived cell patches to the generation of enough cells for a nucleic acid analysis. Gene editing efficiency was derived from the deconvoluted Sanger sequencing data performed by DECODR, ICE and/or TIDE. The use of these bioinformatic software to quantify and determine the nature of gene edits from Sanger sequencing is in concordance with results from next-generation sequencing analyses [19,24,25]. 

The nature of gene edits was also analysed with DECODRE, ICE and/or TIDE using data from Sanger sequencing, as described above. In the RNP-transfected ASK-1 cell, the pattern of edit was +1, +2, −1, −2, −5, −6 and −15 bp edits, with the +1 bp constituting 50% of the total edit types (Supplementary Figure S1). A deletion of up to 15 bp, as observed in ASK-1, has not previously been reported in RNP-edited salmonids cell lines, although short indels of ±1 to ±6, characteristic of the NHEJ repair pathway, have been reported [19,24]. For CHSE-214 cells transfected with RNP, and SHK-1 cells transfected with px459 where the *cr2* gene was targeted, the predominant edit types were −1 and −4 bp, respectively, indicating that the nature of edits is cell-type dependent. Further, the edit pattern also varied between single cells of the same cell type; this is not unexpected given the randomness of the NHEJ cell repair systems, which is expected to vary from cell to cell. All the described indel mutation types were identified in both the heterogenous FACS-enriched cell collection (in the 24-well plate) and the homogenous clonal single cells derived from 16 randomly selected cells in the FACS-sorted 96-well plate, confirming that the single homogenous cell collections are subsets of the heterogenous cell collection. A 77 bp insert, with 80% identity to the *hnRNP R* (heterogeneous nuclear ribonucleoprotein R) gene of A. salmon, was also identified in 31 experimental samples following sequencing of the target regions of the *cr2* gene in the CHSE-214 cell (Supplementary Figure S2a). Fragments of the sgRNA and a PAM site were found flanking the target region, suggesting that the *hnRNP R* is an off-target site for the sgRNA, and that a DSB would have occurred in this site leading to the insertion of the 77 bp insert. It was tempting to attribute this event to an insertion mutation, given that a similar phenomenon was previously reported in the RNP-edited RTgutGC salmonid cell line [24]. Since this is a rare event in an NHEJ repair pathway, numerous samples from the SHAM non-transfected control cells were further sequenced to confirm this. However, the 77 bp insert was identified in six control samples, which indicated that the insertion was not a result of a CRISPR/Cas9-induced mutation. Given the importance of this event, all samples (both experimental and control) in which the 77 bp insert was identified were re-analysed, first by 2% agarose gel purification before being re-subjected to a Sanger sequencing analysis. The 77 bp insert was absent in all the re-analysed samples, leading to the conclusion that the insertion was due to ligation, into the cloning vector, of an amplicon from a non-specific primer amplification sequel to Sanger sequencing. 

### 2.4. Successful Clonal Expansion of ASK-1, CHSE-214 and SHK-1 Cells Following FACS Enrichment and Antibiotic Selection

**Figure 4:**
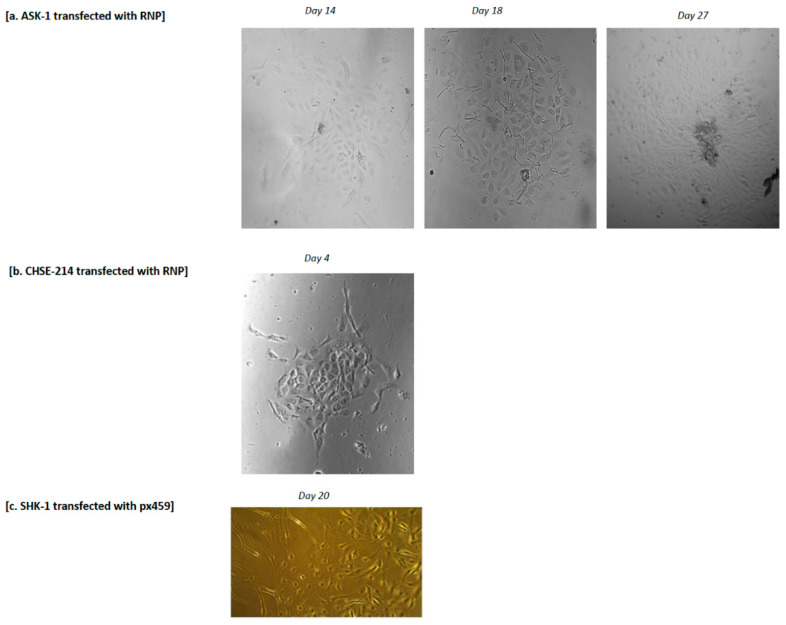
Isolation and clonal expansion of single cell lines following FACS or puromycin selection of positively transfected cells. (**a**) Micrographs of FACS-isolated single cell-derived clonal ASK-1 cell line at day 14, day 18 and day 27 following FACS (magnification 20× for day 14 and day 18; 5× for day 27); (**b**) micrograph of FACS-isolated single cell-derived clonal CHSE-214 cell line at day 4 after FACS (magnification 10×); (**c**) micrograph of puromycin-selected SHK-1 cells at day 20 after puromycin treatment (magnification 10×).

The isolation and expansion of clonal cell lines from a mixed population of transfected cells is paramount for a high level of gene editing, as well as for phenotypic characterization of mutant cells. Single RNP-transfected cells incubated for at least 4 days post electroporation were isolated by FACS into 96-well plates containing conditioned media. The conditioned media consisted of equal volumes of fresh and spent media supplemented with 20% FBS without antibiotics. Similarly, surviving cells of the px459-transfected cells following puromycin exposure were incubated in conditioned media. Patches of cells derived from single cells were detected after 4, 14 and 20 days for CHSE-214, ASK-1 and SHK-1 cells, respectively (Figure 4). 

For the SHK-1 cells, it was not possible to determine whether the cell patches were derived from a single cell following puromycin treatment, as serial dilution was not possible with antibiotic selection. Immediately after puromycin treatment of the px459 transfected SHK-1 cells, no cell was visible in both the non-transfected control and the treated wells; however, after 20 days of incubation of both the control and treated wells, a patch of cells was observed in the treated wells but not in the control wells. Step-by-step propagation, expansion and frozen cell stocks were successfully achieved for ASK-1 and SHK-1 cells, but not for the CHSE-214 cell, which was lost to bacteria contamination. 

## 3. Discussion

The current study achieved clonal cell isolation of edited salmonids cells following the efficient gene editing of ASK-1, SHK-1 and CHSE-214 using the RNP and plasmid CRISPR/Cas9 strategies. The ability to isolate and clonally expand single cells harbouring a specific mutation is paramount for high-level gene editing; the phenotypic characterization of mutant cell lines; understanding gene functions related to, for example, disease resistance; and investigation of biosafety-related issues such as off-targets of the CRISPR/Cas technology. Conducting gene editing in vitro in cell lines before in vivo trials is necessary to prioritize effective candidate genes and to reveal whether a knockout will lead to adverse effects, thus, affecting fish welfare. The present study is part of a project aimed at systematically identifying the resistance genes of A. salmon to the infectious salmon anaemia virus (ISAV) using CRISPR/Cas9, and to identify the potential biosafety-related challenges of the technology. To establish this platform, the *cr2* and *mmp9* genes were randomly selected as model genes from lists of genes previously reported to be associated with the ISAV infection of A. salmon [26,27]. ASK-1, SHK-1 and CHSE-214 are the most commonly used salmonids cell lines, but they are characterized by slow growth, poor transfection efficiency, difficult single cell clonal isolation and expansion [28]. In the current study, high transfection efficiency (90–95%) of the sgRNA:Cas9 complex by electroporation was achieved for the three cell lines. This is the second report of such high transfection efficiency via electroporation of salmonids cells (the first report being the recent publication by Gratacap et al. [19]), thus, verifying the reported high transfection efficiency [19] of these hitherto difficult-to-transfect salmonids cells. The obtained high transfection is remarkable given that transfection is key to high gene editing efficiency, and salmonids cells—including ASK-1, SHK-1 and CHSE-214—are intrinsically difficult to transfect [28] due to the low incubation temperature (15–22 °C) and high saturation of the phospholipids in the cellular membrane compared to mammalian cells [29]. The comparative low transfection efficiency (1–5%) by the plasmids (px458 and px459) can be attributed to a difference in cargo, because the low transfection efficiency of salmonids cells by plasmids (both by electroporation and chemical mediated transfection) has generally been reported [30,31]. Two studies [30,31] previously reported up to 40% positive plasmid electroporation, although the plasmids used in these studies were not adapted for CRISPR/Cas gene editing in contrast to px458 and px459, which, in addition to sgRNA, also encoded the GFP and puromycin markers, respectively. Transfection is, thus, an important parameter to be optimized in the use of the plasmid-mediated CRISPR/Cas strategy for the gene editing of fish cell lines that grow at low temperatures. 

Notwithstanding the low transfection of the plasmids compared to the RNP, both strategies achieved high gene editing efficiency targeting the *cr2* and *mmp9* genes in the three salmonids cells, although a 5% editing efficiency was recorded for a few samples from the CHSE-214 cells transfected with *cr2* RNP complex. Together with the observed variability across cell lines and between single cell clones within a cell line, the present study indicated that both gene efficiency and gene edit pattern are dependent on the cell line, target region and NHEJ DNA repair mechanism of the individual cell. Similar observation of the dependency of gene edit pattern and efficiency on the cell line, target region and cellular repair of the DSB of an individual cell has been reported in previous studies in RTgutGC cell line [24] and RTG-2, ASK-1, SHK-1 and CHSE-214 cell lines [19]. The RTgutGC cell recorded a very low editing efficiency [24] compared to the high editing efficiency in ASK-1, SHK-1 and CHSE-214 cells in the present study and the study by [19], and these differences can be attributed to the characteristics of the cell lines. Target region was also reported to have impacted gene editing efficiency [19] as a result of differences in chromatin accessibility between different genomic regions [32]. Further, the observed differences in edit pattern within the same cell line when different gene loci, i.e., *cr2* and *mmp9*, were targeted showed that the NHEJ repair mechanism can vary between individual cells, which is expected given the randomness of the NHEJ system. Gene editing efficiency in ASK-1 and CHSE-214 cell lines was higher in cells that were incubated for 7 and 9 days, compared to cells incubated for 4 days post electroporation, which can be attributed to the slower activity of the Cas9 protein at the lower temperature at which the salmonids cells were incubated, thus, requiring a longer time to achieve gene editing in comparison to mammalian cells [33]. Similar delayed gene editing activity of Cas9 and Cas12a in fish cells grown at a reduced temperature has been reported [19]. Cas9 is sensitive to temperature, and reduced activity has been reported at temperatures below 37 °C in several mammalian cell lines [34]. In addition, cleaved DNA product is strongly bound to the protein at low temperatures [35]. Further, the expression of sgRNA encoded by plasmids is temperature dependent, with higher expression at a high temperature [34]. Thus, the development of a Cas9 variant with optimal activity at the low incubation temperature of salmonids cells, as well as optimizing sgRNA vector expression systems, can increase the editing efficiency of the cells.

Ultimately, the establishment of clonal lines of edited single cells is required for high-level gene editing, especially for sites with low editing efficiency. The present study achieved, for the first time, the isolation of gene-edited single cells of A. salmon-derived ASK-1 and SHK-1. Clonal cells from the single-edited CHSE-214 cell line were also achieved in the present study, verifying the report of [20], which, until now, was the only published report of a successful clonal isolation of single-edited CHSE-214 cells. A previous unsuccessful attempt at the clonal isolation of SHK-1 following RNP-based CRISPR/Cas9 gene editing and FACS enrichment was reported [19]. A similar unsuccessful attempt at clonal isolation of RTgutGC following FACS enrichment of RNP gene-edited cells was also reported [24], although the same study reported success using an old difficult-to-reproduce cloning cylinder. The differences in the methods of the present study compared with previous studies [19,24] with unsuccessful attempts may be due to (i) the absence of antibiotics from the present study; (ii) the incubation of cells for seven days (and longer) post electroporation and prior to FACS enrichment; (iii) the incubation of isolated single cells for a longer period of 14 days for ASK-1, 20 days for SHK-1 and 4 days for CHSE-214, or until clonal cells were detected. Salmonid cells generally have low tolerance to antibiotics and other types of stress; thus, the absence of antibiotics in the media used in the present study, in contrast to the use of antibiotics in a previous study [19], may explain the successful clonal isolation of the gene-edited cells. Longer incubation of the edited cells immediately after electroporation and prior to FACS enrichment could also be an important factor because in the previous unsuccessful attempt at the single cell isolation of gene-edited RTgutGC cells where antibiotics were also not used in the media [24], the cells were incubated only for 2 days post electroporation and FACS enrichment. Longer incubation after electroporation allows cell recovery from the stress of transfection before being subjected to another round of stress due to FACS. In fact, our experiment showed greater success in isolating single cells from cells incubated for 14 and 21 days after electroporation, compared to cells incubated for 4- and 7-days post electroporation. Thus, the absence of antibiotics, the use of conditioned media with a high percentage of FBS, and a longer incubation time clearly contributed to the success of the clonal cell isolation and expansion of the edited cells in this study. Further optimization of the method is required to increase the growth rate as well as reduce the time required for clonal cell isolation. 

The current study was limited by bacteria/fungal contamination during the period of clonal isolation, due to the required long incubation time for cell growth. This can be circumvented in future studies by the gradual introduction of antibiotics/antifungi into the media after approximately 7 days of FACS enrichment, or once the first patch of clonal cells becomes visible. Other pitfalls related to the CRISPR/Cas9 gene editing of salmonids cells include challenges related to poor annotations (e.g., gene names duplication and poor sequence quality) of existing published genomes, which leads to difficulty in sgRNA design, as many genes sequences are not curated in major databases, e.g., the NCBI (National Centre for Biotechnology Information) database; false assessment of sgRNA’s off-targets; primer non-specific binding; and the non-amplification of several genes by designed primers. For example, the 77 bp insert identified in this study—which was due to the non-specific amplification of a non-target region—could have erroneously been regarded as gene editing resulting from the applied CRISPR/Cas9 strategy. Further, the challenge of a gene duplication event in A. salmon makes the selection of trait-related gene targets difficult, and multiple copies of the same gene in the genome challenges phenotypic testing because the function of a knocked-out gene can easily be replaced by another gene in a different locus in the genome. Overall, the current study, especially the optimized method of the clonal isolation and expansion of gene-edited single cells, provides an excellent platform for high throughput and highly efficient gene editing. This will enable the direct testing of specific phenotypes of interest, leading to a better understanding of the genetics and immunology of the commercially important A. salmon in particular, and salmonids fish species in general. 

## 4. Materials and Methods

### 4.1. Cell Culture

The following cell lines were used in this study: (i) Atlantic salmon kidney 1 (ASK-1), obtained from the Federal Research Institute for Animal Health, Germany; (ii) salmon head kidney 1 (SHK-1) cells, a gift from the Norwegian Veterinary Institute, Oslo; and (iii) Chinook salmon embryo 214 (CHSE-214) cells, obtained from Sigma-Aldrich, United Kingdom. Both ASK-1 and SHK-1 are adherent immortalized cell lines from Atlantic salmon (A. salmon *Salmo salar*); the CHSE-214 is an adherent immortalized cell line from Chinook salmon (*O. tshawytscha*). Vials of frozen cells taken from liquid nitrogen were thawed in the warm (70 °C) for less than 1 min and aseptically transferred into a T-25 cm tissue culture flask containing cell culture medium. The culture medium consisted of Leibovitz L15 (Gibco, NY, USA) supplemented with 10% foetal bovine serum (FBS) (Biowest, Nuaillé, France) without antibiotics and CO_2_. The cells were incubated in the dark (flasks were wrapped in aluminium foil) at 22 ± 0.1 °C until >75% confluency was achieved. The ASK-1 and SHK-1 cells were split 1:2, while CHSE-214 was split 1:3 at confluency. All the cells were passaged at least three times before being used in an experiment.

### 4.2. Design, Synthesis and In Vitro Cleavage Evaluation of sgRNA

**Figure 5:**
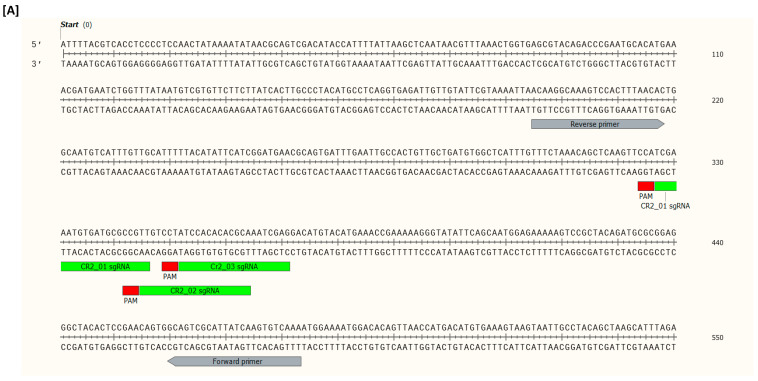

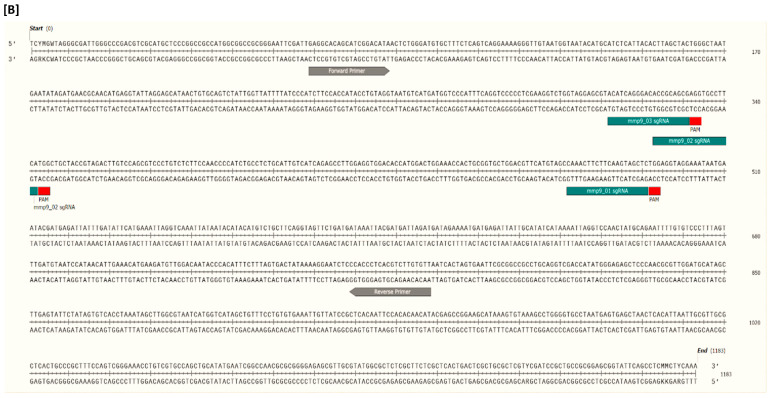
Targeted regions of interest for (**A**) *cr2* and (**B**) *mmp9* indicated by their respective sgRNAs and flanked by primers used in amplifying these regions.

This study is part of a larger project aimed at systematically identifying the genes of A. salmon that are responsible for the susceptibility of the fish to ISA(V); thus, the two genes *cr2* (encoding the complement receptor type 2) and *mmp9* (encoding matrix metalloproteinase 9) were selected as targets to establish the CRISPR/Cas9 platform within the project. Both genes have been associated with A. salmon susceptibility to ISA(V) [26,27]. The design of the sgRNAs was performed using the CRISPR Design Tool (https://design.synthego.com/#/; Synthego Inc., Menlo Park, CA, USA; accessed 23 May 2022) based on the *Salmo salar* RefSeq ICSASQ V2 published genome sequence. Three sgRNAs that targeted different sections of each gene were designed. Details of all sgRNAs together with the primers used to amplify the sgRNA target regions are given in Table 1 and Figure 5. 

The synthesis of sgRNA was performed using the EnGen^®^ sgRNA synthesis kit, *S. pyogenes* (New England Biolabs, UK), in accordance with the manufacturer’s instructions. Briefly, a target-specific oligo was first designed, in which the sgRNA sequence oligos were appended to the 5′ end of the T7 promoter sequence, *TTCTAATACGACTCACTATA*, and a 14-nucleotide overlap sequence, *GTTTTAGAGCTAGA* (overlapped with *S. pyogenes* Cas9 scaffold oligo) was appended to the 3′ end, such that the sgRNA oligo, which is preceded with a ‘G’ at the 5′ end (N_20_ in the target-oligo sequence), was in the middle of the target-specific oligo, i.e., the sgRNA was flanked by the T7 promoter sequence and the overlap sequence: TTCTAATACGACTCACTATAG[*N*]_20_GTTTTAGAGCTAGA. The synthetic sequence of the designed target-specific oligos was supplied by Invitrogen (MA, USA). The laboratory synthesis of the sgRNA was carried out by mixing the target-specific oligos with the manufacturer’s (New England Biolabs, Hitchin, UK) reaction mix, comprising nuclease-free water (2 μL); EnGen 2× sgRNA reaction mix (dNTPs, *S. pyogenes* Cas9 scaffold oligo) (10 μL); target-specific DNA oligo (1 μM; 5 μL); DTT (0.1 M; 1 μL); and EnGen sgRNA Enzyme mix (2 μL). The mixture was incubated at 37 °C for at least 1 h. The resulting sgRNA contained the target-specific crRNA sequence as well as the tracrRNA. To ensure optimal activity, the synthesized sgRNA was purified using the Monarch RNA clean-up kit (New England Biolabs, Hitchin, UK) to remove proteins, salts and unincorporated nucleotides. The concentration of the resulting sgRNA was measured using the Nanodrop 2000c (ThermoFisher Scientific, MA, USA). An aliquot of each sgRNA was denatured (incubated at 65 °C for 5 min) and analysed on 2% agarose gel to confirm their sizes. All sgRNAs were stored at −80 °C until used or no later than 3 months. 

The activities of the synthesized sgRNAs were evaluated using an in vitro cleavage assay system. The cells in wells were lysed with 200 μL of lysis buffer containing 20 μL of proteinase K. Genomic DNA (gDNA) was extracted with the GenElute^TM^ Mammalian Genomic DNA Miniprep kit (Sigma, MI, USA) according to the manufacturer’s instructions. The sgRNA target regions of *cr2* and *mmp9* were PCR-amplified using 20 ng of gDNA in a 50 μL reaction that consisted of 1 μL of Taq polymerase (Sigma, MI, USA); 5 μL of 10× buffer; 1 μL of dNTP; 2.5 μL of each of the forward and reverse primers (Table 1); and 37 μL of H_2_O with 30 cycles of amplification. For the cleavage assay, the generated amplicon (300 ng) served as substrate in a reaction mixture that consisted of 600 ng of sgRNA; 1000 ng of Cas9-GFP (Sigma, MI, USA); 5.5 μL of 10× NEB buffer (New England Biolabs, Hitchin, UK); 1.5 μL of 10× BSA (New England Biolabs, Hitchin, UK); and 15 μL of nuclease-free water. As controls, the same reaction mixtures, but containing no sgRNA and Cas9, were set up in parallel. The reaction mixtures were incubated at 37 °C for 1 h 30 min. To denature excess RNA, 1 μL of RNAase was added to the mixtures and Incubated at 37 °C for 15 min, followed by the addition of 1 μL of STOP solution (30% glycerol, 1.2% SDS, 250 mM EDTA (pH 8.0)) and additional incubation at 37 °C for 15 min. Cleaved substrates were analysed on 2% agarose gel. 

### 4.3. RNP Complex Formation

The RNP complexes were formed by mixing sgRNA and Cas9-EGFP (Sigma-Aldrich, Hamburg, Germany) in a 1:1, 3:1 and 9:1 sgRNA:Cas9-EGFP ratio in nuclease-free 1× OPTIMEM (Gibco, MA, USA). The mixture was incubated at room temperature for 10 min and kept on ice until used or stored at −20 °C for no more than three months. For simplicity, Cas9-EGFP is written as Cas9 and was used for all RNP or Cas9 control transfections in this study.

### 4.4. Plasmid Construction

To assemble the sgRNA plasmids, the complementary oligonucleotides (forward and reverse oligos; see Supplementary Table S1) of the 20-nucleotide target sequence were flanked by the BbsI sites. The oligos were phosphorylated and annealed using T4 polynucleotide kinase (NEB, UK) in 10× T4 ligation buffer (NEB, UK) and cycling-incubated at 37 °C for 30 min, 95 °C for 5 min, and ramped down to 25 at 5 °C per min^−1^. The annealed products were ligated into pX458 (pspCas9(BB)-2A-GFP (a gift from Feng Zhang; Addgene plasmid ID: 48138) and pX459 (pspCas9 (BB)-2A-Puro V2.0 (gift from Feng Zhang; Addgene plasmid ID: 62988) sgRNA expression vectors using T4 DNA ligase (New England Biosciences, Hitchin, UK) and incubated at 25 °C for 2 h, as previously described [36]. The two expression vectors, px458 and px459, respectively, encoded EGFP and puromycin as selection markers.

### 4.5. Transfection of ASK-1, SHK-1 and CHSE-214 Cells by Electroporation with RNP and Plasmid

The cells at approximately 80% confluency were trypsinized, detached and washed in PBS at 100× *g* and resuspended in Opti-MEM at 10^7^ cells/mL. For the RNP transfection, 4 μL of the complexed RNP (1:1, 3:1 or 9:1 sgRNA:Cas9 ratios) at a final concentration of 5 μM was added to 10 μL of 10^7^ cells/mL. Electroporation of the cells was carried out using the Neon Transfection system (Invitrogen, MA, USA). For plasmid transfection, 3 μg of px458 or px459 was added to 10 μL of 10^7^ cells/mL. Electrical pulse at 1600 V, 10 ms, for 3 pulses for RNP electroporation, or at 1200 V, 20 ms, for 2 pulses for plasmid electroporation were delivered to the RNP/cells or plasmid/cells mixtures in a 10 μL Neon tip, in accordance with the manufacturer’s instructions, but with Opti-MEM instead of Neon R Buffer (Invitrogen, MA, USA). The electroporated cells were dispensed into 12-well plates containing 1 mL of conditioned L15 media (equal volumes fresh media and spent media plus 20% FBS) without antibiotics in order to facilitate cell recovery. The controls consisted of (i) sham-electroporated cells, i.e., cells electroporated with Opti-MEM resuspension medium, (ii) cells electroporated with Cas9 protein, or (iii) empty plasmid (plasmid with an empty sgRNA scaffold, i.e., with no ligated sgRNA sequence). All the described experimental conditions were set up in triplicate wells and triplicate plates and incubated in the dark at 22 °C with no CO_2_. The cells were checked for viability and fluorescence after 24 h by fluorescence microscope (ZEISS AX10 vert.A1) and further incubated for 2–14 days for the px458 and RNP-electroporated cells, or until >75% confluency for the px459 electroporated cells. 

### 4.6. T7 Endonuclease 1 (T7E1) Mismatch Detection Assay

The in vivo cleavage ability of the sgRNAs was performed on cells transfected with the RNP complex using the T7E1 assay in two stages. In the first stage, the targeted regions in the *cr2* and *mmp9* genes were PCR-amplified using the primers (*cr2*: F-CACTTGCCCTACATGCCTCA, R-GCCACAACAACCTCATCCCA; *mmp9*: F -TATGTCCGATGCTGTGCCTC; R: AACACAAGACGTGAGGGTGG), yielding DNA amplicons of approximately 487 bp for *cr2* and 710 bp for *mmp9*. The PCR products were purified using the NucleoSpin Gel and PCR clean-up kit (Machery-Nagel, Germany). To determine whether indel mutations were present, heteroduplexes were formed by denaturing (98 °C for 5 min) and re-annealing (95–85 °C at −2 °C/s; 85–25 °C at −0.1 °C/s) the PCR product. The annealed heteroduplexes were incubated in endonuclease 1 in a 1:19 enzyme:substrate ratio at 37 °C for 15 min, in accordance with the manufacturer’s instructions. Thereafter, 1 μL of proteinase K was added to the mixture and incubated at 37 °C for 5 min to inactivate the T7 endonuclease 1 activity. A fragment analysis was performed by electrophoresis of the mixture in a 2% agarose gel to provide an estimate of the in vivo cleavage of the target regions by the RNP complexes.

### 4.7. Enrichment and Propagation of Edited Cells

Following incubation for 4 to 14 days for RNP- or 2 days for pX458-electroporated cells, the transfected cells were enriched via FACS on the BD FACSAria III cell sorter (BD, NJ, USA). The trypsinized pelleted cells were washed in PBS by centrifuging at 100× *g* for 5 min. The cells were fully resuspended and filtered through a 35 μm cell strainer cap (Falcon, MA, USA) in order to remove cell clumps. Cell sorting was carried out using a 130 μm nozzle. Single-cell events were gated, and the percentage of GFP-positive cells together with the intensity of GFP fluorescence from each cell was measured. GFP-positive cells were sorted into a 96-well plate for single-cell colony expansion or into a 24-well plate containing 200–500 μL of conditioned media and incubated in the dark at 22 °C. For the px459-transfected cells (and non-transfected control cells) at over >75% confluency, growth media was replaced with complete L15 media containing 0.36 μg/μL of puromycin and incubated in the dark at 22 °C; plates were observed, and the media changed every 4 days or until all the cells in the control wells were dead. The puromycin-containing L15 media was then replaced with conditioned media in the plates containing transfected cells incubated at 22 °C until confluency. 

### 4.8. Sanger Sequencing and CRISPR Edit Analysis

To determine the efficiency of gene editing and the exact type of gene edits, the targeted regions in the *cr2* and *mmp9* genes (Table 1, Figure 1) were amplified using gDNA extracted from transfected and non-transfected control cells, as described in Section 4.2, and purified either by gel extraction or NucleoSpin PCR clean up (Machery-Nagel, Düren, Germany), followed by PCR amplicon Sanger sequencing. Sanger sequencing was performed on cells from both the 96-well and 24-well plates. Briefly, 7 μL of purified PCR product was ligated into 1 μL (50 ng) of pGEM-t Easy Vector System (Promega, USA), in accordance with the manufacturer’s instructions. Transformation of the ligated product was carried out using the JM109 High Efficiency Competent Cells (Promega, WI, USA), according to standard protocol. Thereafter, 200 μL of transformed culture was plated onto an LB/ampicillin/IPTG/X-Gal plate and incubated overnight at 37 °C. The plates were observed after overnight incubation for blue/white colonies; white colonies generally contain desired inserts. DNA was extracted from the pelleted overnight culture of single bacteria colonies picked from both treated and control plates and grown in LB ampicillin-containing broth at 37 °C. Gel-purified DNA served as template for PCR amplification used for Sanger sequencing according to the following protocol: The samples were diluted to 100 ng/μL, and 5 μL was mixed in a PCR tube with a sequencing master mix which contained (i) big dye, (ii) sequencing buffer, and (iii) M13 primer. The samples were then amplified at 34 cycles (cycling temperature and duration: 96 °C at 30 s; 50 °C at 15 s; 60 °C at 4 s). The amplified products were submitted to the sequencing platform of the University of North Norway Teaching Hospital (https://unn.no/fag-og-forskning/forskning/dna-sekvensering#utfyllende-informasjon-pa-engelsk--detailed-information-in-english), last accessed 23 May 2022. An assessment of the nature and frequency of the CRISPR edits was conducted using the following free online bioinformatic tools: Deconvolution of Complex DNA Repair (DECODR; https://decodr.org/analyze; last accessed 23 May 2022); Inference of CRISPR Edits (ICE; https://ice.synthego.com; last accessed 23 May 2022); and Tracking of Indels by Decomposition (TIDE; http://shinyapps.datacurators.nl/tide-batch/; last accessed 23 May 2022). 

## Figures and Tables

**Table 1:** Single guide RNA sequences and primers used for amplification and sequencing of target genomic regions.

CRISPR/Cas9 Target	sgRNA Sequences (5′-3′)	Primers (5′-3′)	Product Size (bp)
*cr2* (exon 2)	cr2_01: AACGGCGCAUCACAUUUCGA cr2_02: UGCGUGUGUGGAUAGGACAA cr3_03: CUCGAUUUGCGUGUGUGGAU	F: TTTGACACTTGATAATGCGACTGC R: ACAAGGCAAAGTCCACTTTAACAC	289
*mmp9* (exon 2)	mmp9_01: CAAACUUCUUCAAGUAGCUC mmp9_02: ACCGCAGCGAGGUGCCUUCA mmp9_03: ACAUCAGGGACACCGCAGCG	F: TATGTCCGATGCTGTGCCTC R: AACACAAGACGTGAGGGTGG	710

## Data Availability

Not applicable.

## Supplementary Materials

The following supporting information can be downloaded at: https://www.mdpi.com/article/10.3390/ijms232416218/s1.
