# Supplementary material for: CRISPR/Cas9-Mediated Gene Editing in Salmonids Cells and Efficient Establishment of Edited Clonal Cell Lines
Source: Int J Mol Sci. 2022 Dec 19;23(24):16218. doi: 10.3390/ijms232416218 (PMC9787932; doi:10.3390/ijms232416218)
Supplement: Supplementary file 1 [file ijms-23-16218-s001.zip › ijms-2029485-supplementary.pdf]

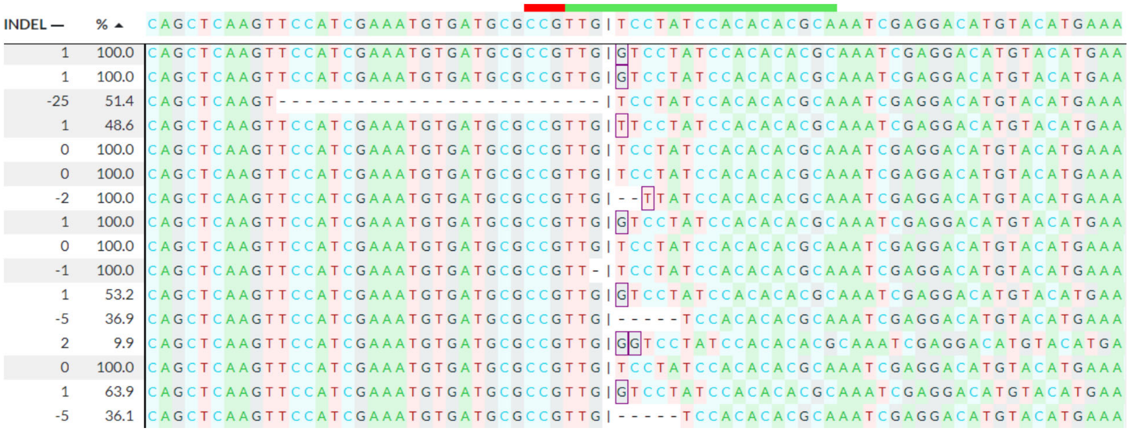

Figure S1. Representative output of DECODRE analysis of an RNP ASK-1 cell. Indel type and editing frequency are indicated (left part of the ouput) as well as the aligned sequences of the wild-type and edited samples (right part of the output). The sgRNA including the PAM sequences (red and green bar) highlights the exact target sequence and Cas9 cut site.

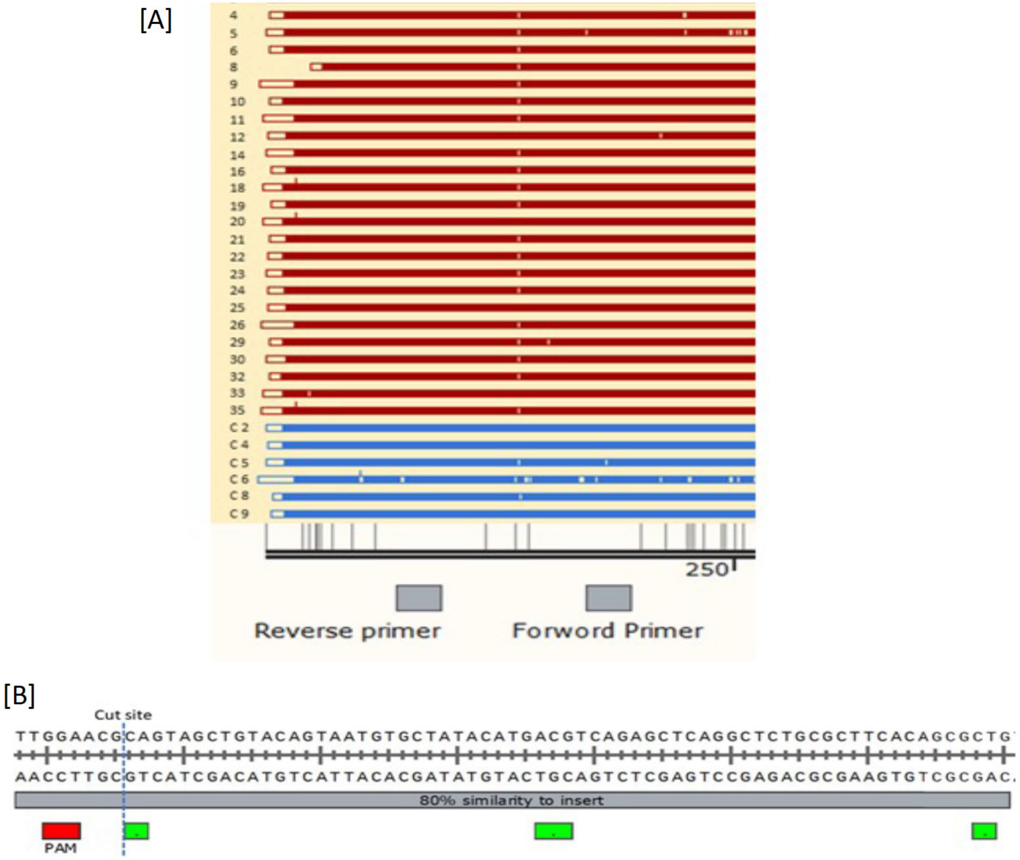

Figure S2. Alignment of the sequences (77 bp insert) that were preferentially incorporated in place of the target region. [A] Blue bars denote control sequence (6 samples); red bars denote the 77 bp insert (31 samples). [B] Alignment of the 77 bp insert with a homologous region, the *hnRNP R* (heterogeneous nuclear ribonucleoprotein R) of A. salmon. The 77 bp insert has high homology (80%) to the *hnRNP R*.

Fragments of the sgRNA (green block) and a PAM site (red block) were found flanking the region, suggesting the *hnRNP R* is an off-target site for the sgRNA, and that a DSB would have occurred in this site leading to the insertion of the 77 bp insert. However, the same insert was found in some control samples, which leads to the conclusion that the insert was not the result of a CRISPR/Cas9-induced mutation

Table S1. Complementary oligonucleotides of respective sgRNA used in cloning of px458 and px459

**Target Gene**	**sgRNA Sequence**	**Complementary oligonucleotides with BbsI sites (highlight)**
cr2	AACGGCGCAUCACAUUUCGA	F: CACCAACGGCGCATCACATTTCGA
		R: AAACTCGAAATGTGATGCGCCGTT
	UGCGUGUGUGGAUAGGACAA	F: CACCTGCGTGTGTGGATAGGACAA
		R: AAACTTGTCCTATCCACACACGCA
	CUCGAUUUGCGUGUGUGGAU	F: CACCCTCGATTTGCGTGTGTGGAT
		R: AAACATCCACACACGCAAATCGAG
mmp9	CAAACUUCUUCAAGUAGCUC	F: CACCCAAACTTCTTCAAGTAGCTC
		R: AAACGAGCTACTTGAAGAAGTTTG
	ACCGCAGCGAGGUGCCUUCA	F: CACCACCGCAGCGAGGTGCCTTCA
		R: AAACTGAAGGCACCTCGCTGCGGT
	ACAUCAGGGACACCGCAGCG	F: CACCACATCAGGGACACCGCAGCG
		R: AAACCGCTGCGGTGTCCCTGATGT
